# Protein-protein interaction network of celiac disease 

**Published:** 2016

**Authors:** Mona Zamanian Azodi, Hassan Peyvandi, Mohammad Rostami-Nejad, Akram Safaei, Kamran Rostami, Reza Vafaee, Mohammadhossein Heidari, Mostafa Hosseini, Mohammad Reza Zali

**Affiliations:** 1 *Basic and Molecular Epidemiology of Gastrointestinal Disorders Research Center, Research Institute for Gastroenterology and Liver Diseases, Shahid Beheshti University of Medical Sciences, Tehran, Iran*; 2 *Hearing Disorders Research Center, Shahid Beheshti University of Medical Sciences, Tehran, Iran*; 3 *Gastroenterology and Liver Diseases Research Center, Research Institute for Gastroenterology and Liver Diseases, Shahid Beheshti University of Medical Sciences, Tehran, Iran*; 4 *Proteomics Research Center, Shahid Beheshti University of Medical Sciences, Tehran, Iran*; 5 *Department of Gastroenterology, Milton Keynes University Hospital United Kingdom*; 6 *Faculty of Medicine, Iran University of Medical Sciences, Tehran, Iran*

**Keywords:** Protein-protein interaction, Network, celiac disease, hub-bottleneck

## Abstract

**Aim:**

The aim of this study is to investigate the Protein-Protein Interaction Network of Celiac Disease.

**Background:**

Celiac disease (CD) is an autoimmune disease with susceptibility of individuals to gluten of wheat, rye and barley**. **Understanding the molecular mechanisms and involved pathway may lead to the development of drug target discovery**.** The protein interaction network is one of the supportive fields to discover the pathogenesis biomarkers for celiac disease.

**Material and methods:**

In the present study, we collected the articles that focused on the proteomic data in celiac disease. According to the gene expression investigations of these articles, 31 candidate proteins were selected for this study. The networks of related differentially expressed protein were explored using Cytoscape 3.3 and the PPI analysis methods such as MCODE and ClueGO.

**Results:**

According to the network analysis Ubiquitin C, Heat shock protein 90kDa alpha (cytosolic and Grp94); class A, B and 1 member, Heat shock 70kDa protein, and protein 5 (glucose-regulated protein, 78kDa), T-complex, Chaperon in containing TCP1; subunit 7 (beta) and subunit 4 (delta) and subunit 2 (beta), have been introduced as hub-bottlnecks proteins. HSP90AA1, MKKS, EZR, HSPA14, APOB and CAD have been determined as seed proteins.

**Conclusion:**

Chaperons have a bold presentation in curtail area in network therefore these key proteins beside the other hub-bottlneck proteins may be a suitable candidates biomarker panel for diagnosis, prognosis and treatment processes in celiac disease.

## Introduction

 Celiac disease (CD) is an autoimmune disease in susceptible individuals sensitive to gluten component of wheat, rye and barley (1, 2), which is responsible for immune reaction response (3). During initiation and development of CD, both genetic (human leukocyte antigen (HLA) genes (DQ2 or DQ8)) and environmental factors (gluten) are implicated participated (4, 5). Celiac patients are at the risk of nutritional deficiency leading to conditions like osteoporosis and iron deficiency anemia (6). 

The proteomics study has been used to identify proteins or changes in protein expression of CD to discover a biomarkers using several techniques, such as antibody and tissue arrays, two-dimensional (2D) gel electrophoresis, mass spectrometry. Results of these techniques are suggesting that we may approached new insight in some diagnostic biomarkers by observing body fluids with posttranslational modifications during signaling (7) which will provide an understanding of the pathogenesis of CD. One of the logical fields to biomarker discovery is to study protein interaction networks, which can be useful because of making scientific abstraction based on principle roles of proteins in the biological function. However, the analysis of protein –protein interactions (PPIs) enable us to better understanding of biological processes, organization, and functions of proteins. In addition, it provides protein complex identification, (8) domain-domain interactions, (9) detection of proteins involved in disease pathways, (10) comparison between model organisms and humans (11) and introducing drug targets from network (12). Consequently, this knowledge can be translated and applied into effective diagnostic and therapeutic strategies (13) by identification of drug targets and hubs (proteins with larger number of interactions) (14, 15). Meanwhile proteomics and PPI networks analysis together are powerful tools to determine associated biomarkers with specific pathways and biological functions (16-23). In this study, the enrichment analysis of selected proteins based on the GO and PPI is investigated to introduce some related molecular biomarkers (as a panel) for celiac disease. 

## Material and Methods

PubMed (PubMed Central), ISI of web knowledge, and Google scholar were searched for full text articles on the associated keywords "proteomic" and "celiac disease" published until April 2016 (24, 25). Based on results from previous gene expression studies, 31 protein candidates were selected for celiac disease and the PPI network was visualized using the Cytoscape 3.3 software (26). "Cytoscape is an open source software project for integrating bimolecular interaction networks with high-throughput expression data and other molecular entity into a unified conceptual state (27). In this study, MINT and Reactome-FLs databases were used for topology visualization. Also, we used Molecular Complex Detection (MCODE) based on topology to find densely connected region to analyze the characteristics of the networks. These include Kappa statistic ≥ 0.5, enrichment and Bonferroni step down method for probability value correction (28). Gene ontology categories were analyzed to identify the function of each highly connected region that was generated by the MCODE. It visualizes the biological terms for large clusters of proteins in a functionally grouped network non-redundantly (29). Functional enrichment for a given cluster was assessed (P-value) quantitatively, using the ClueGO tool (30). 

**Figure 1:**
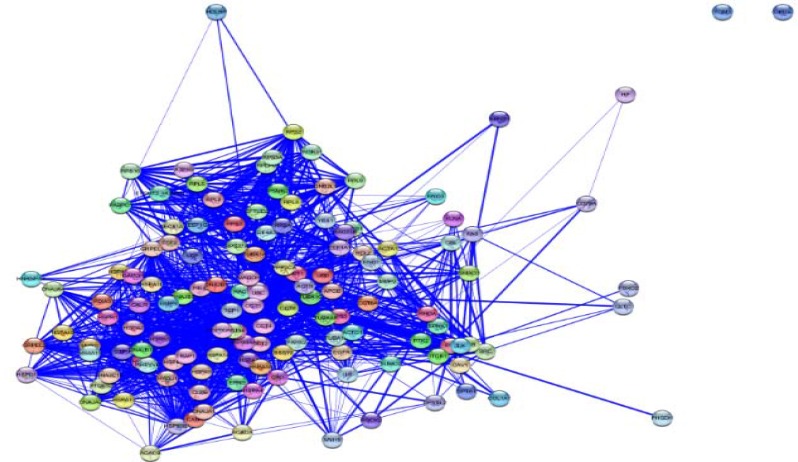
PPI network of celiac disease consists of 134 nodes and 1686 edges

**Table 1:** The proteins with highest degree in celiac that introduce as hub sorted by degree number

stringdb	Gene name	Degree	Betweenness centrally
9606.ENSP00000344818	ubiquitin C	110	0.26405012
9606.ENSP00000335153	heat shock protein 90kDa alpha (cytosolic), class A member 1	79	0.06416467
9606.ENSP00000325875	heat shock protein 90kDa alpha (cytosolic), class B member 1	64	0.03145362
9606.ENSP00000227378	heat shock 70kDa protein 8	56	0.02820046
9606.ENSP00000317334	t-complex 1	53	0.01534998
9606.ENSP00000377958	chaperonin containing TCP1, subunit 4 (delta)	52	0.01440804
9606.ENSP00000299300	chaperonin containing TCP1, subunit 2 (beta)	52	0.01366942
9606.ENSP00000324173	heat shock 70kDa protein 5 (glucose-regulated protein, 78kDa)	51	0.02409187
606.ENSP00000258091	chaperonin containing TCP1, subunit 7 (beta)	51	0.01527081
9606.ENSP00000299767	heat shock protein 90kDa beta (Grp94), member 1	49	0.01383266

## Results

**Figure 2:**
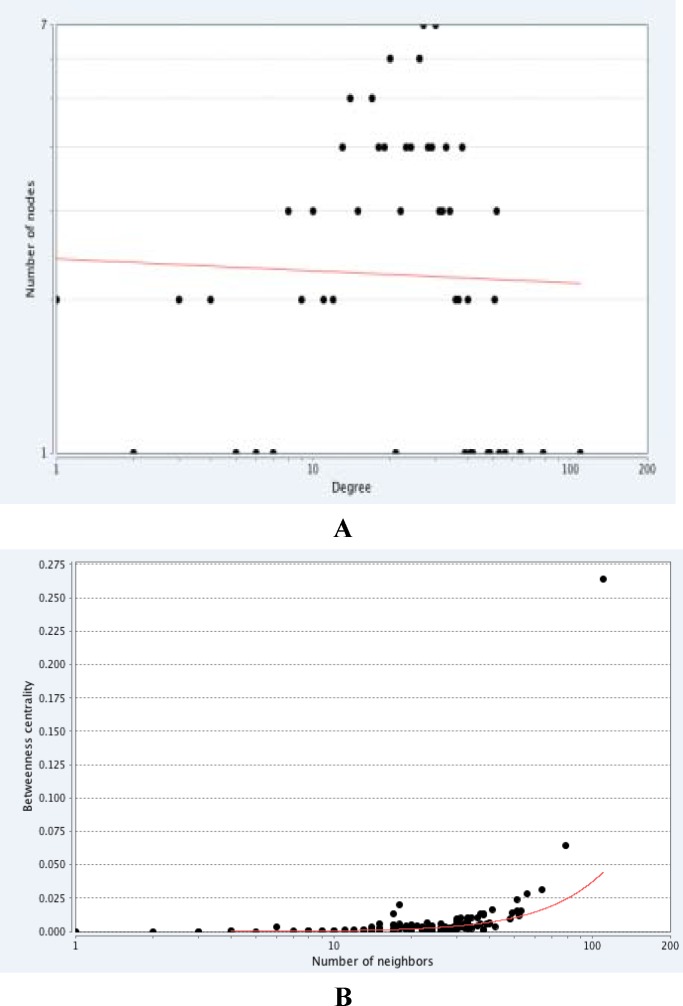
Th power law distribution of nod's centrality measures. (A) Degree distribution of the PPI network in celiac. (B) Betweenness centrality distribution of PPI network in celiac

The topological analyses were carried out via algorithms such as centrality measures. Graph centrality measures like degree, betweenness and closeness centrality are so useful in the identification of nodes that are functionally crucial in the network (31). In the PPI network the nodes with high degree defined as hub proteins and the nodes with high betweenness defined as bottleneck proteins, which both paly a fundumental role in the network (32). Cytoscape analysis revealed a great number of close interconnections that can be seen in Figure 1. The power law of node degree distribution is one of the most important criteria of biological networks which indicated that the PPI networks were scale-free (figure 2). This scale-free distribution network implies on the presence of proteins with high centrality values computed by Network Analyzer in celiac patients. The red line indicates the power law. The R-squared value is computed on logarithmized values, which are equal to 0. 60, - 0.02 and the correlation= 0.86, 0.001 for betweenness centrality and degree, respectively.

**Figure 3:**
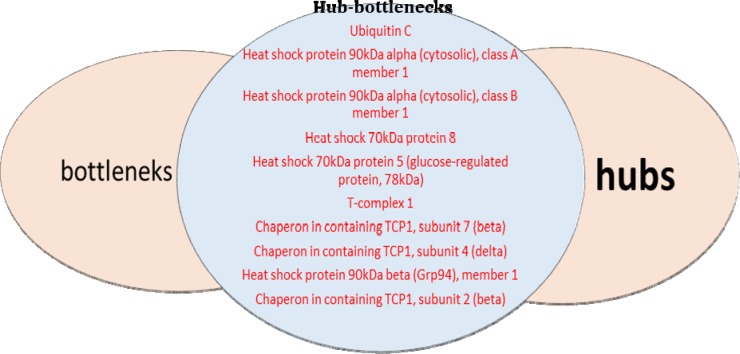
The proteins with high scores of degree as hub and high scores of betweeness in celiac that introduce as bottleneck. The common proteins in hub and bottleneck group introduce as hub- bottleneck

 To identify crutial nodes in PPI networks, two centrality criteria, including degree and betweenness have been used in this study. First, we prepared the list of proteins, which were hubs in PPI network. The results indicate that some nodes have a large number of links to other protein nodes and act as hubs. Then we determine the bottlenecks (highest betweeness nodes) in the network (table 1). It was shown that there are common proteins between hubs and bottleneck (figure 3). 

As represented in table 1, Ubiquitin C, Heat shock protein 90kDa alpha (cytosolic and Grp94); class A, B and 1 member, Heat shock 70kDa protein, and protein 5 (glucose-regulated protein, 78kDa), T-complex, Chaperon in containing TCP1; subunit 7 (beta) and subunit 4 (delta) and subunit 2 (beta), have been introduced as hub – bottleneck. Chaperon in containing TCP1, subunit 2 (beta) have been introduced as hub – bottlenecks. In this study hubs and bottlenecks were the same as hub-bottlenecks in which bold the importance of these proteins. 

The results of MCODE analysis were shown in table 2. Bonferroni step down was applied for p-value adjustment and pathways with adjusted p-value<0.05 were selected. Further analysis of complex by MCODE revealed six sub networks for the network (Table 3). HSP90AA1, MKKS, EZR, HSPA14, APOB and CAD are proteins that involved in protein networks of celiac disease and have been determined as seeds. 

**Figure 4:**
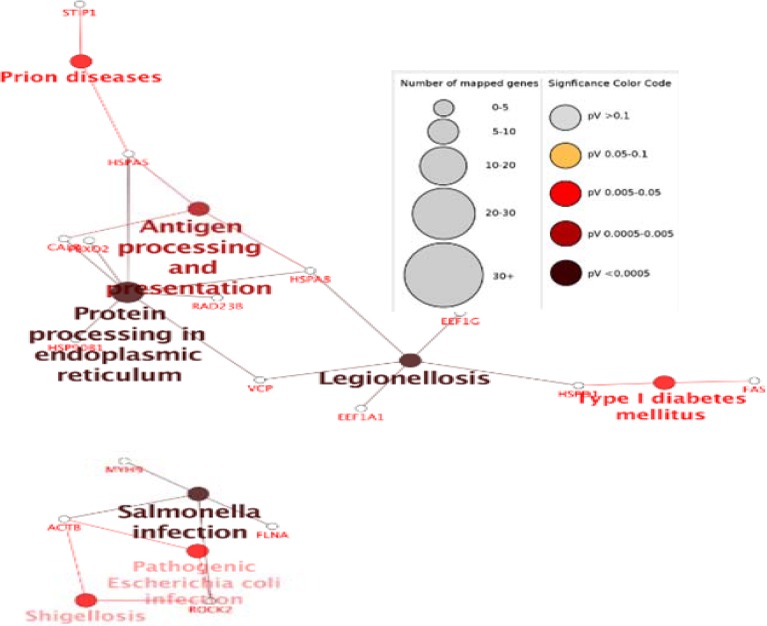
Functional distribution of biological process of modules of celiac disease

Functional distribution of the biological process of celiac modules was shown in figure 4. According to information presented in figure 3 some of the important proteins as hub – bottleneck are involved in antigen processing and presentation (HSPA5, HSPA8), protein processing in the endoplasmic reticulum (HSP90B1, HSPA5, HSPA8) and legionellosis (HSPA8). 

**Table 2:** MCODE algorithm analysis demonstrate clusters and have been sorted by score

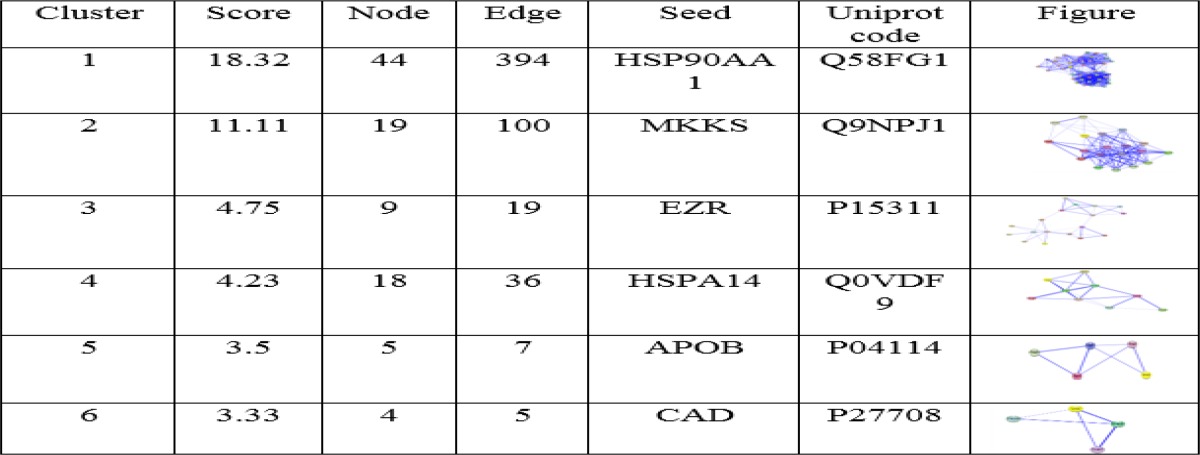

## Discussion

Since proteins act as complex and corporate with each other in cellular events and pathways, their deregulation are resulted in disorders. One way to evaluate and analyze the pathways is molecular mapping (33). Recently, quantitative tools have been developed for analyzing the molecular mapping and networks. Analyzing the network attributes of gene-expression data might reveal the pattern of gene expression in diseases, which might be helpful to identify new potential drug targets (34). Therefore, in this study, the network is analyzed to extract celiac disease markers by Cytoscape software. As represented in table 1; Heat shock proteins and chaperonines have been introduced as a hub – bottleneck. In this study, hubs and bottlenecks were the same as hub-bottlenecks. Therefore, it can be concluded that these proteins play important roles in the pathology of disease. The highest score of degree and centrality belong to Ubiquitin C (UbC) which is one of the four genes encoding ubiquitin in the mammalian genome. The role of this gene in several processes such as oxidative stress, UV irradiation, heat shock, and translational impairment is discussed (35). The 6q21-22 region of ubiquitin-pathways components was confirmed as a celiac disease susceptibility locus (36). Heat shock proteins (HSPs) have been introduced as hub-bottleneck such as Heat shock protein 90kDa alpha (cytosolic), class A and B member, Heat shock 70kDa protein, Heat shock protein 90kDa beta (Grp94), member 1 and Heat shock 70kDa protein 5 (glucose-regulated protein, 78kDa). HSPs are molecular chaperones that increase survival by allowing cells to resist stress through cyto-protective mechanisms (37). Heat shock proteins (HSPs) are classes of proteins that have already appeared as drug targets for autoimmune diseases that protect cells against harmful extracellular factors. HSPs play a key role in the maintenance of epithelial cell structure and function and also put immunomodulatory effects. They are responsible for cell repair processes after damage and adequate protein folding, proliferation and apoptosis, influence the degradation of proteins and modulate cell signaling (38). In this study, Hsp90AA1 has also been introduced as another hub-bottleneck and seed with the highest score. The expression changes of HSP90AA1 in celiac disease is poorly documented, but in cancer research the 90-kDa heat shock protein HSP90AA1 is critical for the stability of several proteins that are important for tumor progression and introduced as a promising target for cancer therapy (39). Introducing HSP 90 AA1 as a drug target and curtail protein in our network is not unexpected because in CD nitric oxide levels increases via gluten consumption and then increases ROS level (40, 41). Interestingly, these oxygen-free radicals induce the expression of various HSPs such HSP 90, which take part in the defense against oxidative stress (42). Another chaperon that in this study introduced as hub-bottelnek was T-complex 1. As mentioned, the chaperonins are key molecular complexes, which are essential in the folding of proteins to stabilization. One member of the chaperonin group of proteins is TCP1, but little is known about this protein in diseases. Studies were shown that both TCP1 beta and TCP1 epsilon are over-expressed in colorectal cancer and indicate a role in colorectal cancer progression (43). It has also been represented alteration in protein expression with chaperone activities such as T-complex protein-1 in a human intestinal epithelial cell line for celiac disease (24). Chaperon in containing TCP1, subunit 7 (beta), Chaperon in containing TCP1, subunit 4 (delta) as well as chaperon in containing TCP1, subunit 2 (beta) are molecular chaperones that are another hub-bottlenecks which also known as members of the chaperonin containing TCP1 complex (CCT). This ATP-dependent complex folds various proteins, including actin and tubulin (44, 45) introduced as a curtail protein in cancer development (46) are considered as novel therapeutic targets in breast cancer (47). Another seed is McKusick-Kaufman/Bardet-Biedl syndromes putative chaperonin (MKKS) as a molecular chaperone that assists the folding of proteins upon ATP hydrolysis. As part of the BBS/CCT complex may play a role in the assembly of BBSome (complex involved in ciliogenesis ) and cytokinesis (48), but there are no obvious documents of its relation with celiac disease up to now. Another seed named Ezrin is the only ERM family member expressed in the intestinal epithelium (49) and interacts with F-actin (50). It has been suggested phosphorylation of Ezrin will regulate the early events in brush border induction (51) and loss of microvilli and brush border lead to common features of several intestinal diseases (52, 53). Another chaperonin that has been suggested as seed is HSPA14. Increased HSP expression in tumour tissues is a common phenomenon (54). The HSPA14 was one of overexpressed proteins in HCC tumour tissues (55). Apolipoprotein B is another seed and a major protein constituent of VLDL (apo B-100), LDL (apo B-100) and chylomicrons (apo B-48). In CD, The basal condition was characterized by low cholesterol absorption, enhanced cholesterol synthesis, and high removal rate of LDL apo B (56). It has also been reported that low high-density lipoprotein-cholesterol concentration associated with CD (57-59). In the celiac disease, protein- protein interaction network the lowest score of seeds was belonged to CAD which encoding four enzymatic activities of the pyrimidine pathway (60). The association of CAD activation and uncontrolled cell proliferation in cancer has been reported(61), but no clear and directed relation has been reported with celiac disease yet. Our major finding pathways- based network analysis between the patients and a normal one, include antigen processing and presentation (HSPA5, HSPA8), protein processing in the endoplasmic reticulum (HSP90B1, HSPA5, HSPA8) and legionellosis (HSPA8). Understanding the molecular mechanisms and involved pathway may lead to the development of drugs target discovery. Through our analysis, it has been provided new insight into celiac pathogenesis by analyzing the networks and consequently presenting the specified pattern of gene expression, which might identify new biomarkers and targets for potential treatment. Introducing chaperons (HSPs &TCP) as powerful biomarkers and drug targets lead to emphasize a possible close molecular relationship between celiac and oxidative stress, repair processes after damage and protein folding. This information can suggest a strong possibility to design drug through suppressing ROS and free radicals. To reveal the possible role(s) of these proteins in celiac disease, further investigations are needed.
